# Longitudinal Speech Outcomes in Foreign-Born and Native-Born Children in Sweden with Cleft Palate, and Influence of Palatal Surgery Timing

**DOI:** 10.1177/10556656261434727

**Published:** 2026-03-23

**Authors:** Louise Lendt, Magnus Becker, Ketty Andersson, Kristina Klintö

**Affiliations:** 1 Division of Speech Language Pathology, Phoniatrics and Audiology, Department of Clinical Sciences in Lund, 5193 Lund University, Lund, Sweden; 2 Division of Speech Language Pathology, Department of Otorhinolaryngology, 59565 Skåne University Hospital, Malmö, Sweden; 3 Division of Surgery, Department of Clinical Sciences in Malmö, 5193 Lund University, Malmö, Sweden; 4 Division of Plastic and Reconstructive Surgery, 59565 Skåne University Hospital, Malmö, Sweden

**Keywords:** cleft palate, cleft lip and palate, speech, surgery, internationally adopted, foreign-born

## Abstract

**Objective:**

To compare longitudinal speech outcomes at ages 5 and 10 years in foreign-born and Swedish-born children with cleft palate with or without cleft lip (CP ± L), and assess impact of cleft type, additional diagnosed conditions, sex, and age at completed primary palatal surgery.

**Design:**

Longitudinal, registry-based cohort study.

**Setting:**

Regional public care university hospitals in Sweden.

**Participants:**

723 children (143 foreign-born, 580 Swedish-born) with CP ± L.

**Interventions:**

Primary and secondary palatal surgery, and speech-language therapy.

**Main outcome measures:**

Dichotomized outcomes of velopharyngeal competence (VPC), percentage of consonants correct (PCC), and non-oral speech errors, and received secondary palatal surgery and speech-language therapy.

**Results:**

A significantly lower proportion of foreign-born than Swedish-born 5-year-olds had VPC (75% vs. 85%, *p* = .007), age-appropriate consonant production (25% vs. 57% *p* < .001), and no non-oral speech errors (67% vs. 84%, *p* < .001). Speech improved longitudinally, yet a significantly lower proportion of foreign-born children had VPC (86% vs. 94%, *p* = .002), age-appropriate consonant production (52% vs. 76%, *p* < .001), and no non-oral errors (82% vs. 93%, *p* < .001) at 10 years. The difference in VPC at age 5 and in all speech outcomes at age 10 was associated with higher age at primary surgery in foreign-born children. They also had higher rates of secondary palatal surgery (*p* < .001) and speech-language therapy (*p* < .001) than Swedish-born peers.

**Conclusions:**

Later primary palatal surgery in foreign-born children was associated with poorer speech outcomes at 10 years. Early primary palatal surgery and continued follow-up are essential to optimize speech development.

## Introduction

Children with cleft palate with or without cleft lip (CP ± L) can have speech difficulties despite early palatal closure. These difficulties are often related to velopharyngeal dysfunction, defined as insufficient closure between the oral and nasal cavities during speech. Velopharyngeal dysfunction may manifest as hypernasality, nasal air leakage, and/or weak articulation.^1,2^ In addition, some children develop compensatory articulatory strategies.^2–4^ These speech characteristics may reduce intelligibility and, in turn, negatively affect peer relations.^5–7^

To date, most research on speech outcomes in children with CP ± L carried out globally has focused on children born in the country where the research was carried out. However, foreign-born children constitute an important group that clinicians regularly encounter in cleft care, both internationally and in Sweden, although their numbers vary over time. Understanding speech development in this group is important because it may differ from that of native-born children due to variations in early care and timing of interventions.^8–10^ Migration and international adoption are global phenomena. Children born abroad with unoperated cleft lip and palate (CLP) represent a group encountered in cleft care across many countries. Sweden's national, standardized cleft care and long-term follow-up provide a unique opportunity to study speech outcomes and development in foreign-born children with CP ± L. These findings are relevant and potentially generalizable to comparable international contexts.

### Speech in Native-Born Children with CP ± L

Speech difficulties in native-born children with CP ± L have been documented across ages. Several studies have reported speech difficulties at the age of 5 years^3,11–15^ and 10 years.^16–20^ Longitudinal research has shown improvements in articulation up to the age of 10 years in children with unilateral CLP (UCLP)^18,21–23^ and in those with cleft palate without cleft lip (CP).^20^ Similarly, velopharyngeal function has been found to improve up to the age of 10 years in children with UCLP,^18,21–23^ in children with CP,^20^ and in those with CP ± L.^24^ However, a proportion of children continue to experience persistent articulation difficulties and/or velopharyngeal dysfunction at 10 years of age. For example, Nyberg et al^22^ reported that among 10-year-olds with UCLP, 30% had distorted /s/ production, 4.4% had retracted oral articulation, and 1.4% showed glottal articulation. Furthermore, in a study by Lohmander et al,^23^ 38% of children with UCLP had velopharyngeal dysfunction at 10 years of age, including cases with mild velopharyngeal impairment. Other studies have reported lower rates of velopharyngeal dysfunction. Nyberg et al^22^ reported 7% of 10-year-olds with UCLP to have mild to severe incompetent velopharyngeal function. In another study by Nyberg et al^20^ 8% of 10-year-olds with CP had moderately incompetent velopharyngeal function. Furthermore, in a study based on the Swedish CLP registry, 4% of 10-year-olds with CP ± L were perceived as incompetent.^24^ In summary, while longitudinal improvements in articulation and velopharyngeal function are observed up to 10 years of age, a notable proportion of children with CP ± L continue to experience speech difficulties. Reported prevalence varies across studies which partly may be due to differences in inclusion criteria, assessed speech variables, and assessment methods. This complicates direct comparisons.

### Speech in Foreign-Born Children with CP ± L

Foreign-born children represent a heterogeneous group, including both non-adopted and internationally adopted (IA) children. This distinction is important because country of birth, age at arrival, family and language backgrounds may differ between these subgroups.

Previous research on non-adopted foreign-born children with CP ± L is limited. To our knowledge, only one study has included this group when examining speech in foreign-born children.^8^ At 5 years of age, non-adopted foreign-born and IA children combined had significantly lower rates of velopharyngeal competence (VPC), age-appropriate consonant production, and speech without non-oral speech errors compared with Swedish-born peers with CP ± L.^8^

Previous studies including children with UCLP,^25^ UCLP and bilateral CLP (BCLP),^26^ and CP ± L^9,10^ suggest that 5-year-old IA children more frequently demonstrate articulation difficulties^9,25,26^ and velopharyngeal dysfunction^10,26^ compared with non-adopted peers. Differences have been reported in consonant proficiency,^25,26^ substitution,^25^ and/or reinforcement with glottal plosives.^26^ Larsson et al^25^ also found a higher frequency of cleft speech characteristics in 5-year-old IA children with UCLP than in non-adopted peers. In contrast, Okhiria et al^26^ reported no significant differences in cleft speech characteristics between IA and non-adopted children with UCLP and BCLP, and Morgan et al found no significant differences in cleft-related errors—a scoring system capturing characteristic phonological patterns—in children with CP ± L aged 3–9 years.^9^ These variations in findings in cleft speech characteristics may reflect differences in cleft types, ages, inclusion criteria and how speech difficulties were defined and measured across studies.

Longitudinal studies suggest that IA children show improvements in speech over time. In a study by Larsson et al,^27^ IA children with UCLP showed gains in both articulation and VPC between ages 5 years and 7–8 years. Similarly, Okhiria et al^26^ reported improvements in consonant accuracy between ages 5 and 10 years in IA children with UCLP and BCLP. However, in both studies a larger proportion of IA children continued to demonstrate speech difficulties compared with their non-adopted peers. At 7–8 years, IA children with UCLP showed articulation scores below normative data, with a median percentage of consonants correct (PCC) of 79.7%, and 18% continued to demonstrate incompetent velopharyngeal function.^27^ By 10 years of age, the group with IA children with UCLP and BCLP had lower PCC, with 30% performing more than 4 standard deviations (SDs) below the age-appropriate norm, lower rates of VPC, and a higher proportion had cleft speech characteristics compared with non-adopted peers.^26^ Overall, research on foreign-born children, particularly non-adopted children, is limited. Although results for specific speech variables vary across studies, findings suggest that a higher proportion of foreign-born children demonstrate speech difficulties. This highlights the need for increased understanding of their longitudinal speech development.

### Speech in Children with CP ± L and Additional Diagnosed Conditions

Additional diagnosed conditions—such as Robin sequence, syndromes, additional malformations, and/or intellectual disability—frequently co-occur with CP ± L.^28–30^ Reported prevalence varies across studies but is generally highest among children with CP (eg, 43%–52% of cases)^28–30^ and somewhat lower among those with CLP (eg, 28%–37%).^29,30^ While these conditions are not the primary focus of the present study, children with additional conditions were included, and their inclusion may affect speech outcomes. Few studies have investigated speech in children with CP ± L and additional conditions, particularly at school age, and findings have been inconsistent. At 5 years of age, some studies have reported poorer articulation and velopharyngeal function in children with additional conditions^28^ and in those with Robin sequence,^31,32^ while one study found no significant differences between children with CP with and without Robin sequence.^33^ In a recent study, 5-year-olds with cleft of the soft and hard palate who had Robin sequence, additional conditions, or both Robin sequence and additional conditions had significantly lower odds of age-appropriate consonant production compared to those without these conditions.^34^ Furthermore, children with Robin sequence and additional conditions also had lower odds of VPC and no non-oral speech errors.^34^ These findings align with Lendt et al,^8^ who reported lower odds of VPC, age-appropriate consonant production, and no non-oral speech errors in 5-year-olds with CP ± L and additional diagnosed conditions compared to peers without such conditions. Similarly, in one study on speech in children with CP, persistent velopharyngeal dysfunction and glottal articulation at 10 years of age were mostly observed in those with additional diagnosed conditions.^35^

### Aims

Foreign-born children represent a small and heterogeneous group, making them challenging to study. However, access to national registry data covering nearly the complete cohort of Swedish children with CP ± L enables longitudinal investigation and comparison with Swedish-born peers.

This registry-based study aimed to assess longitudinal speech outcomes at ages 5 and 10 years in foreign-born children with CP ± L compared to Swedish-born peers with CP ± L. We further examined the impact of cleft type, additional diagnosed conditions, sex, and age at completed primary palatal surgery on speech outcomes. Finally, we compared the occurrence of secondary palatal surgery and speech-language therapy between the groups.

## Methods

This was a longitudinal registry-based study using data from the Swedish CLP registry. Only data included in the registry were used.

### Data from the Swedish CLP Registry

Data were obtained from the Swedish CLP registry via the Record Centre South, Lund, Sweden. A manual review of all data entries was conducted to ensure consistency and accuracy before statistical analysis.

#### Coverage, Reporting Degree, and Reliability of Speech Data

The registry was established to ensure equal care, evaluate treatment outcomes, and improve treatment methods.^36^ All six Swedish CLP centers contribute data to the Swedish CLP registry on background characteristics of included individuals, surgery, orthodontics and speech, from birth to 19 years. All children receiving cleft care in Sweden, both Swedish-born and foreign-born children, are offered participation. The average degree of national coverage was 95.1% for children born between 2009 and 2018.^36^ The reporting degree for surgery and speech exceeded 90%.^36^ Speech variables, including speech-related quality indicators, have been shown to be reliable,^37–39^ enabling longitudinal studies of speech and intervention in nearly the entire complete cohort of Swedish children with CP ± L. Several previous studies have used cohorts from the Swedish CLP registry, with partially overlapping birth years and participants, as well as differing inclusion and exclusion criteria.^8,13,24,34,40,41^ This should be considered when comparing results across studies.

#### Baseline Data

At the child's first visit to the CLP team, baseline data were registered, including cleft diagnosis according to ICD-10,^42^ whether the child was born in Sweden or abroad, and whether the child was adopted.^36^ Furthermore, whether the child had undergone surgery abroad was recorded as a binary variable. Information on interventions carried out prior to arrival in Sweden, including surgical and speech-language interventions, or medical history were not recorded. The presence of additional diagnosed conditions, that is, syndromes, Robin sequence, and additional malformations, (yes/no) was registered at the first visit and continuously updated during follow-ups. No details on type of syndrome or additional malformations were available. Data on country of birth, age at arrival in Sweden, and pre-arrival family or language environment were not available.

#### Surgical Data

Information on cleft-related surgeries performed in Sweden was continuously registered. Data included operation codes according to the Swedish National Board of Health and Welfare's classification of health interventions,^43^ whether the surgery was primary or secondary, and the child's age at the time of surgery. Currently in Sweden, according to official surgical protocols, children with CP ± L undergo primary palatal surgery either in one stage, between 9 and 14 months of age, or in two stages—beginning with soft palate closure (with or without lip plasty) at around 6 months—followed by hard palate closure at approximately 2 years of age.^13,36^ For a limited period, one CLP center performed palatal closure in three stages in selected cases.^13^ Secondary palatal surgery and/or speech-improving surgery may be performed when required. Information on surgeon's experience and cleft width were not available. Further details on surgical routines and national treatment protocols are described in Klintö et al^13^ and Schaar Johansson et al.^40^

#### Speech Registrations

Children with CP ± L were followed by speech-language pathologists as part of routine multidisciplinary care up to 19 years of age. During these follow-ups, speech data—including ratings of VPC—were routinely entered into the CLP registry by a CLP team speech-language pathologist, based on perceptual assessment of standardized audio recordings.^36^ No retrospective re-assessment of speech was performed for the purpose of this study. The speech assessment followed a structured protocol based on the Swedish Articulation and Nasality Test (SVANTE),^1^ including a single-word test by picture naming, sentence repetition and continuous speech. For single-word naming, either the standard version of SVANTE (59 words) or the restricted word list (30 words), developed for cross-linguistic speech assessment was used.^1^ In a few cases, the word test from the randomized controlled trial Timing of Primary Surgery for Cleft Palate (TOPS) (36 words)^14^ was applied.

All word tests were designed to assess cleft speech characteristics according to the same principles.^44^ The entire word was elicited, but only the target consonant was phonetically transcribed according to the International Phonetic Alphabet,^45^ and used for the analysis of consonant production. Based on these transcriptions, PCC and percentage of non-oral speech errors were calculated.^1^ In PCC, the target consonant was scored as incorrect if realized as another phoneme. Weak articulation and audible nasal air leakage, when marked with diacritics, were scored as correct in the PCC calculation. Non-oral speech errors included glottal and pharyngeal articulations as well as active nasal fricatives. VPC was rated using the 3-point scale VPC-R,^46^ based on impressions of hypernasality, audible nasal air leakage and weak articulation in the whole speech sample. The scale categories were competent/sufficient, marginally incompetent/insufficient and incompetent/insufficient.^1^

For multilingual children, an interpreter, fluent in the child's first language was used during the visit if needed. The single word testing by picture-naming was performed in Swedish. The tests included words with only one or two syllables, which allowed children with limited Swedish proficiency to name the pictures. For a few children, when needed, words were elicited by repetition. As VPC was assessed across the entire speech sample (words, sentence repetition, and conversational speech), the child's first language could be included in the evaluation.

In addition to the speech assessments, the registrations also included information on diagnosed intellectual disability, either established in Sweden by a psychologist or reported from a prior diagnosis before arrival in Sweden, and whether the child had received speech-language therapy since the previous visit (yes/no).^36^ Details on the type and timing of speech-language therapy were not available. Information on the number of speech-language pathologist visits and whether the child had a diagnosed speech or language disorder or hearing impairment were registered. However, due to substantial missing data these variables were not included in the analyses.

### Participants

The study population was identified based on predefined inclusion and exclusion criteria.

Inclusion criteria were children with CP ± L, born between 2009 and 2015, who had at least one speech variable registered in the CLP registry at both 5 and 10 years of age.

Exclusion criteria were deceased children (n = 15) and those whose speech assessments were either registered outside the predefined time frames (5 years ± 6 months and 10 years ± 1 year) or missing at one or both ages (n = 220).

Among the excluded children, 28% were foreign-born, 52% had additional diagnosed conditions, 55% had CP, 28% had UCLP, and 17% had BCLP. The distribution of sex (boys 58%, girls 42%) was similar to that of the included group (Table 1). In total, 723 children (143 foreign-born and 580 Swedish-born) met the inclusion criteria (Table 1). Foreign-born children, defined as being born outside Sweden, had not followed the Swedish cleft care program since birth. This group was heterogeneous and included both IA children and non-adopted foreign-born children, that is, children born outside Sweden who were not IA. Regardless of whether they were non-adopted or IA, these children may have differed in several other aspects, such as country of birth, age at arrival in Sweden, access to cleft care, language background and parental country of birth, information that was not captured in the registry.

**Table 1.** Characteristics of Participants and Timing of Completed Primary Palatal Surgery.

Variable	Foreign-born	Swedish-born	*p*-value	Effect size
Variable, n (%)	143 (19.8)	580 (80.2)		
Cleft palate (cleft of the soft palate and cleft of the soft and hard palate)	16 (11.2)	314 (54.1)	<.001	0.344
Unilateral cleft lip and palate	88 (61.5)	178 (30.7)		
Bilateral cleft lip and palate	39 (27.3)	88 (15.2)		
Boys	98 (68.5)	316 (54.5)	.002	0.113
Girls	45 (31.5)	264 (45.5)		
Internationally adopted	126 (88.1)			
Surgery abroad	96 (67.1)	1 (0.1)		
Additional diagnosed conditions	23 (16.1)	160 (27.6)	.005	0.105
Age of completed primary palatal surgery^a^			<.001	0.426
<10 months	1 (1)	123 (21.2)		
>10 to ≤13 months	4 (3.8)	136 (23.5)		
>13 to ≤18 months	8 (7.7)	71 (12.3)		
>18 to ≤25 months	26 (25)	134 (23.1)		
>25 to ≤36 months	43 (41.3)	102 (17.6)		
>36 months	22 (21.1)	13 (2.2)		
Secondary palatal surgery 0–5 years	43 (30.1)	85 (14.7)	<.001	0.161
Secondary palatal surgery 5–10 years	47 (32.9)	94 (16.2)	<.001	0.167
Secondary palatal surgery 0–10 years	73 (51)	150 (25.9)	<.001	0.217
Speech-language therapy 0–5 years	101 (70.6)	260 (45)	<.001	0.205
Speech-language therapy 5–10 years	80 (56.3)	202 (34.9)	<.001	0.175
Speech-language therapy 0–10 years	113 (79)	321 (55.3)	<.001	0.193

Pearson χ^2^-test comparisons between foreign-born and Swedish-born children with frequency, percentage, and associated *p*-value and effect size (Cramér's V).

a Data were missing for 40 children who had undergone completed primary palatal surgery abroad.

Children with missing data were excluded from respective analyses but included in all analyses where data were available. For each speech variable, only data from children with registrations at both 5 and 10 years for that specific variable were included. Analyses involving age at primary palatal surgery required available data on primary palatal surgery performed in Sweden, as information on surgery performed abroad was not available in the registry. Thus, children with no data on primary palatal surgery performed in Sweden were excluded in these analyses but included in all analyses not involving age at primary palatal surgery.

### Speech Outcomes and Categorical Change Variables

For statistical analysis, speech variables were dichotomized into speech-related quality indicators. These quality indicators were used because they have demonstrated reliability^37–39^ and have been employed in previous Swedish CLP registry studies.^8,13,24,34^ VPC was defined as competent or marginally incompetent velopharyngeal function, which generally does not lead to recommendations for surgical intervention, whereas incompetent velopharyngeal function often does. This quality indicator has demonstrated reliability at both 5^38^ and 10 years of age.^37^ Age-appropriate consonant production was defined as performance within 2 SDs below the mean of normative data for non-cleft children, corresponding to ≥86% correct consonants at 5 years and ≥92.3% at 10 years of age.^1^ This quality indicator has demonstrated reliability at both 5 years^39^ and 10 years of age.^37^ No non-oral speech errors were defined as <5%, allowing for a small margin of error. This quality indicator has also been shown to be reliable at both ages.^37,38^

For each quality indicator, a categorical change variable was created to capture longitudinal patterns of speech development between the ages of 5 and 10 years. Each change variable included four categories: stable poor outcome (poor speech outcomes at both ages), deteriorated outcome (good speech outcome at 5 years, poor speech outcome at 10 years), improved outcome (poor speech outcome at 5 years, good speech outcome at 10 years), and stable good outcome (good speech outcomes at both ages). By recoding the data using these change variables, statistical dependence between the two time points was reduced, facilitating longitudinal analysis of speech development.

### Intervention Variables

The CLP registry provided data on whether the children have received speech-language therapy or not. It also provided data on number of speech-language therapy sessions; however, the reporting degree for this variable was insufficient. Details of the therapy, such as type, intensity or duration, were not registered. Thus, the CLP registry data only provided data for a basic descriptive binary variable (yes/no), indicating whether the child had received speech-language therapy. This was assessed within three age intervals: 0–5, 5–10, and 0–10 years of age.

Age at completed primary palatal surgery was categorized into six groups: ≤10 months, >10 to ≤13 months, >13 to ≤18 months, >18 to ≤25 months, >25 to ≤36 months, and >36 months. The first five categories correspond to previous Swedish CLP registry studies,^8,24,40^ while the sixth category (>36 months) was added to capture children who underwent later primary palatal surgery, a pattern more common among those born abroad.^8^ These categories reflect time intervals associated with an increased risk of secondary palatal surgery^47^ or poorer speech outcomes.^3,13,48,49^ Secondary palatal surgery was analyzed as a binary variable (yes/no), including re-operation of previously operated palatal structures and/or speech-improving surgery (eg, pharyngeal flap, buccal flap, sphincter pharyngoplasty or fistula closure) within three age intervals: 0–5 years of age, 5–10 years of age and 0–10 years of age.

### Statistical Analysis

Pearson's chi-squared test was used to compare participant characteristics between foreign-born and Swedish-born children and to analyze group differences in the longitudinal development of the three change variables (VPC change, consonant production change and non-oral speech errors change). The variables included in the statistical analysis were: foreign-born or Swedish-born, cleft type, sex, additional diagnosed conditions, age at primary palatal surgery, dichotomized speech outcomes (VPC, age-appropriate consonant production, and no non-oral speech errors), secondary palatal surgery, speech-language therapy, and longitudinal changes in speech outcomes.

Differences in speech outcomes at 5 and 10 years between foreign-born and Swedish-born children were analyzed using binary logistic regression models. Odds ratios (ORs) for positive speech outcomes, with corresponding 95% confidence intervals (CIs) were reported. Three models were applied: a univariable analysis (Model A); a multivariable model adjusted for cleft type, sex, and additional diagnosed conditions (Model B); and a third multivariable model in which age at completed primary palatal surgery was added as a potential mediator (Model C). Data on age at completed primary palatal surgery were only available for children who underwent primary palatal surgery in Sweden. Consequently, Model C excluded children with no available data on this variable, whereas Models A and B included all children with available speech outcome data.

All analyses were conducted using SPSS Statistics version 29 (IBM Corp., Armonk, NY, USA). A two-tailed *p*-value of <0.05 was considered statistically significant. The choice of statistical methods was made in consultation with a statistician.

## Results

### Participant Characteristics

Among the foreign-born children (n = 143), 126 were IA and 17 were non-adopted (Table 1). Ninety-six of these children had undergone unspecified cleft-related surgery abroad (partial or complete).

The foreign-born group had a significantly higher proportion of boys (69% vs 55%, *p* = .002) and a significantly lower proportion of additional diagnosed conditions (16% vs 28%, *p* = .005), including Robin sequence, syndromes, additional malformations, and/or intellectual disability, compared with the Swedish-born group (Table 1). The distribution of cleft types differed significantly between the groups (*p* < .001). BCLP (27% vs 15%) and UCLP (62% vs 31%) were significantly more common among foreign-born children, whereas CP was significantly more prevalent among Swedish-born children (11% vs 54%) (Table 1).

### Palatal Surgery and Speech-Language Intervention

Data on primary palatal surgery were available for 683 children, with 40 missing cases (39 foreign-born and 1 Swedish-born) due to completed surgery abroad. Foreign-born children had undergone completed primary palatal surgery at significantly higher ages than their Swedish-born peers (Table 1). Completed primary palatal closure after 25 months was more common among foreign-born (62%) than Swedish-born children (20%), whereas closure before 18 months was less common among foreign-born than Swedish-born children (13% vs 57%). Secondary palatal surgery was performed significantly more often in foreign-born children before 5 years (30% vs 15%) and between 5 and 10 years (33% vs 16%). Overall, 51% of foreign-born children had undergone secondary palatal surgery by the age of 10 years, compared with 26% of Swedish-born peers.

Data on speech-language therapy were available for 721 children, with two missing entries at 0–5 years (both Swedish-born) and two at 5–10 years (1 foreign-born and 1 Swedish-born). Speech-language therapy was significantly more common among foreign-born children before 5 years (71% vs 45%), between 5 and 10 years (56% vs 35%), and between 0–10 years of age (79% vs 55%) than among Swedish-born children (Table 1).

### VPC

Data on VPC were available for 714 children, with 9 missing cases (3 foreign-born and 6 Swedish-born). In Model C, 40 children had missing data (39 foreign-born and 1 Swedish-born) due to having undergone only primary surgery abroad. In the univariable analyses, foreign-born children had significantly lower odds of VPC compared with Swedish-born children at 5 years (OR = 0.543, 95% CI = 0.348–0.848) and at 10 years (OR = 0.413, 95% CI = 0.232–0.738) (Model A, Table 2). In multivariable logistic regression models adjusted for cleft type, sex, and additional diagnosed conditions (Model B), the difference remained significant at both 5 years (OR = 0.522, 95% CI = 0.321–0.849) and 10 years (OR = 0.437, 95% CI = 0.232–0.823) (Table 2). However, after further adjustment for age at completed primary palatal surgery, as shown in Model C, the difference was no longer significant at either 5 or 10 years.

**Table 2.** Binary Logistic Regression Modeling Velopharyngeal Competence in Foreign-Born versus Swedish-Born Children at 5 and 10 Years of Age.

Variable	Model A 5y OR (95% CI) N = 714	Model B 5y OR (95% CI) N = 714	Model C^a^ 5y OR (95% CI) N = 674	Model A 10y OR (95% CI) N = 714	Model B 10y OR (95% CI) N = 714	Model C^a^ 10y OR (95% CI) N = 674
Foreign-born (ref: Swedish-born)	**0.543** (0.348–0.848)	**0.522** (0.321–0.849)	0.757 (0.413–1.385)	**0.413** (0.232–0.738)	**0.437** (0.232–0.823)	0.555 (0.261–1.182)
Boy (ref: Girl)	–	1.055 (0.697–1.598)	1.052 (0.677–1.635)		0.875 (0.486–1.577)	0.748 (0.417–1.475)
Cleft type (ref: UCLP)						
CP	–	1.047 (0.633–1.733)	0.774 (0.434–1.381)		0.933 (0.446–1.952)	0.567 (0.246–1.307)
BCLP	–	0.725 (0.42–1.25)	0.67 (0.373–1.202)		**0.443** (0.222–0.887)	**0.394** (0.187–0.832)
Additional diagnosed conditions (ref: No)	–	**0.506** (0.326–0.785)	**0.499** (0.311–0.8)		0.748 (0.397–1.409)	0.692 (0.351–1.365)
Age at completed primary palatal surgery (ref: <10 months)						
>10 to ≤13 months	–	–	0.784 (0.357–1.722)			0.573 (0.162–2.032)
>13 to ≤18 months	–	–	0.857 (0.335–2.195)			1.206 (0.208–7.004)
>18 to ≤25 months	–	–	0.606 (0.275–1.334)			0.643 (0.174–2.38)
>25 to ≤36 months	–	–	**0.321** (0.148–0.695)			**0.172** (0.052–0.568)
>36 months	–	–	**0.257** (0.09–0.735)			**0.11** (0.027–0.452)

Univariable model (A), and multivariable models (B, C) adjusted for other variables presented in each column. Odds ratios (ORs) with 95% confidence intervals (CIs). Statistically significant OR values are marked in bold. Ref, reference category; UCLP, unilateral cleft lip and palate; CP, cleft palate; BCLP, bilateral cleft lip and palate.

a Fewer children were included in Model C at both ages, due to missing data on age at completed primary palatal surgery for children with completed surgery abroad.

Completed primary palatal surgery after 25 months was associated with lower odds of VPC at both ages, as indicted in Model C. At 5 years, additional diagnosed conditions were also associated with lower odds (OR = 0.499, 95% CI = 0.311–0.8). At 10 years, children with BCLP had lower odds of VPC compared to children with UCLP (OR = 0.394, 95% CI = 0.187–0.832) (Model C, Table 2).

### Age-Appropriate Consonant Production

Data on age-appropriate consonant production were available for 687 children, with 36 missing cases (13 foreign-born and 23 Swedish-born). In Model C, 34 additional children had missing data (33 foreign-born and 1 Swedish-born) due to having undergone surgery only abroad. Univariable analyses showed that foreign-born children had significantly lower odds of age-appropriate consonant production at 5 years (OR = 0.249, 95% CI = 0.162–0.384) and 10 years (OR = 0.351, 95% CI = 0.236–0.521) compared with Swedish-born peers (Model A, Table 3). These differences remained significant in the multivariable models adjusted for cleft type, sex, and additional diagnosed conditions at both 5 years (OR = 0.349, 95% CI = 0.219–0.556) and 10 years (OR = 0.546, 95% CI = 0.353–0.845) (Model B, Table 3). After further adjustment for age at completed primary palatal surgery, as shown in Model C, foreign-born children remained less likely to demonstrate age-appropriate consonant production at 5 years (OR = 0.391, 95% CI = 0.221–0.691), but the difference was no longer significant at 10 years (Table 3).

**Table 3.** Binary Logistic Regression Modeling Age-Appropriate Consonant Production in Foreign-Born versus Swedish-Born Children at 5 and 10 Years of Age.

Variable	Model A 5y OR (95% CI) N = 687	Model B 5y OR (95% CI) N = 687	Model C^a^ 5y OR (95% CI) N = 653	Model A 10y OR (95% CI) N = 687	Model B 10y OR (95% CI) N = 687	Model C^a^ 10y OR (95% CI) N = 653
Foreign-born (ref: Swedish-born)	**0.249** (0.162–0.384)	**0.349** (0.219–0.556)	**0.391** (0.221–0.691)	**0.351** (0.236–0.521)	**0.546** (0.353–0.845)	0.678 (0.401–1.147)
Boy (ref: Girl)	–	**0.677** (0.482–0.95)	**0.679** (0.476–0.969)		**0.631** (0.428–0.931)	**0.654** (0.435–0.984)
Cleft type (ref: UCLP)						
CP	–	**2.235** (1.507–3.316)	**1.574** (0.999–2.48)		**3.418** (2.114–5.528)	**2.473** (1.433–4.27)
BCLP	–	**0.426** (0.26–0.7)	**0.374** (0.221–0.633)		**0.415** (0.264–0.654)	**0.349** (0.216–0.566)
Additional diagnosed conditions (ref: No)	–	**0.534** (0.359–0.794)	**0.558** (0.368–0.847)		**0.482** (0.308–0.756)	**0.467** (0.29–0.753)
Age at completed primary palatal surgery (ref: <10 months)						
>10 to ≤13 months	–	–	**0.537** (0.292–0.987)			0.871 (0.379–2.004)
>13 to ≤18 months	–	–	0.712 (0.354–1.433)			0.641 (0.263–1.558)
>18 to ≤25 months	–	–	**0.433** (0.234–0.803)			0.634 (0.283–1.423)
>25 to ≤36 months	–	–	**0.265** (0.14–0.501)			**0.374** (0.167–0.837)
>36 months	–	–	**0.314** (0.119–0.833)			**0.318** (0.111–0.913)

Univariable model (A), and multivariable models (B, C) adjusted for other variables presented in each column. Odds ratios (ORs) with 95% confidence intervals (CIs). Statistically significant OR values are marked in bold. Ref, reference category; UCLP, unilateral cleft lip and palate; CP, cleft palate; BCLP, bilateral cleft lip and palate.

a Fewer children were included in Model C at both ages, due to missing data on age at completed primary palatal surgery for children with completed surgery abroad.

Several variables were associated with lower odds of age-appropriate consonant production at both ages, including additional diagnosed conditions at 5 years (OR = 0.558, 95% CI = 0.368–0.847) and 10 years (OR = 0.467, 95% CI = 0.29–0.753), BCLP (compared with UCLP) at 5 years (OR = 0.374, 95% CI = 0.221–0.633) and 10 years (OR = 0.349, 95% CI = 0.216–0.566), boys (compared with girls) at 5 years (OR = 0.679, 95% CI = 0.476–0.969) and 10 years (OR = 0.654, 95% CI = 0.435–0.984), and completed primary palatal surgery after 25 months (Model C, Table 3). Conversely, children with CP showed higher odds of age-appropriate consonant production compared to those with UCLP at both 5 years (OR = 1.574, 95% CI = 0.999–2.48) and 10 years (OR = 2.473, 95% CI = 1.433–4.27). At 5 years, completed primary palatal surgery after 18 months or between 10 and 13 months (OR = 0.537, 95% CI = 0.292–0.987) was also associated with lower odds (Model C, Table 3).

### Non-Oral Speech Errors

Data on non-oral speech errors were available for 692 children, with 31 missing cases (11 foreign-born and 20 Swedish-born). In Model C, an additional 34 children were missing (33 foreign-born and 1 Swedish-born) due to having undergone surgery only abroad. In the univariable analyses, foreign-born children had significantly lower odds of no non-oral speech errors compared with their Swedish-born peers at 5 years (OR = 0.368, 95% CI = 0.251–0.593) and 10 years (OR = 0.328, 95% CI = 0.189–0.569) (Model A, Table 4). After adjusting for cleft type, sex, and additional diagnosed conditions, these differences remained significant at 5 years (OR = 0.443, 95% CI = 0.278–0.706) and 10 years (OR = 0.333, 95% CI = 0.18–0.618) (Model B, Table 4). However, further adjustment for age at completed primary palatal surgery, as shown in Model C, showed that the difference persisted at 5 years (OR = 0.525, 95% CI = 0.301–0.916) but was no longer significant at 10 years (Table 4).

**Table 4.** Binary Logistic Regression Modeling No Non-Oral Speech Errors in Foreign-Born versus Swedish-Born Children at 5 and 10 Years of Age.

Variable	Model A 5y OR (95% CI) N = 692	Model B 5y OR (95% CI) N = 692	Model C^a^ 5y OR (95% CI) N = 658	Model A 10y OR (95% CI) N = 692	Model B 10y OR (95% CI) N = 692	Model C^a^ 10y OR (95% CI) N = 658
Foreign-born (ref: Swedish-born)	**0.386** (0.251–0.593)	**0.443** (0.278–0.706)	**0.525** (0.301–0.916)	**0.328** (0.189–0.569)	**0.333** (0.18–0.618)	0.497 (0.231–1.1071)
Boy (ref: Girl)	–	**0.628** (0.41–0.962)	**0.608** (0.387–0.954)		0.748 (0.417–1.341)	0.63 (0.335–1.186)
Cleft type (ref: UCLP)						
CP	–	1.228 (0.735–2.051)	0.737 (0.411–1.32)		0.753 (0.35–1.621)	0.501 (0.211–1.192)
BCLP	–	**0.523** (0.317–0.865)	**0.474** (0.277–0.812)		**0.271** (0.138–0.533)	**0.239** (0.113–0.503)
Additional diagnosed conditions (ref: No)	–	0.642 (0.405–1.019)	**0.584** (0.354–0.964)		0.609 (0.329–1.127)	0.548 (0.281–1.068)
Age at completed primary palatal surgery (ref: <10 months)						
>10 to ≤13 months	–	–	0.86 (0.325–2.275)			1.151 (0.322–4.115)
>13 to ≤18 months	–	–	**0.327** (0.125–0.855)			0.47 (0.134–1.647)
>18 to ≤25 months	–	–	**0.301** (0.125–0.728)			0.955 (0.276–3.299)
>25 to ≤36 months	–	–	**0.232** (0.095–0.563)			0.333 (0.107–1.034)
>36 months	–	–	**0.127** (0.043–0.377)			**0.138** (0.037–0.523)

Univariable model (A), and multivariable models (B, C) adjusted for other variables presented in each column. Odds ratios (ORs) with 95% confidence intervals (CIs). Statistically significant OR values are marked in bold. Ref, reference category; UCLP, unilateral cleft lip and palate; CP, cleft palate; BCLP, bilateral cleft lip and palate.

a Fewer children were included in Model C at both ages, due to missing data on age at completed primary palatal surgery for children with completed surgery abroad.

Lower odds of no non-oral speech errors were associated with BCLP (compared with UCLP) at both 5 years (OR = 0.474, 95% CI = 0.277–0.812) and 10 years (OR = 0.239, 95% CI = 0.113–0.503). Completed primary palatal surgery after 13 months was associated with lower odds of no non-oral speech errors at 5 years, while surgery after 36 months was associated with lower odds at 10 years (OR = 0.138, 95% CI = 0.037–0.523). Boys (compared with girls) (OR = 0.608, 95% CI = 0.387–0.954) and additional diagnosed conditions (OR = 0.584, 95% CI = 0.354–0.964) were associated with lower odds at 5 years but not at 10 years (Model C, Table 4).

### Longitudinal Speech Development

Speech outcomes improved longitudinally from 5 to 10 years in both groups. The proportion of children with VPC increased from 75% to 86% in the foreign-born group and from 85% to 94% in the Swedish-born group (Figure 1). The proportion of children with age-appropriate consonant production increased from 25% to 52% in the foreign-born group and from 57% to 76% in the Swedish-born group. Similarly, the proportion of children with no non-oral speech errors increased from 67% to 82% among the foreign-born group and from 84% to 93% among the Swedish-born group (Figure 1).

**Figure 1.**
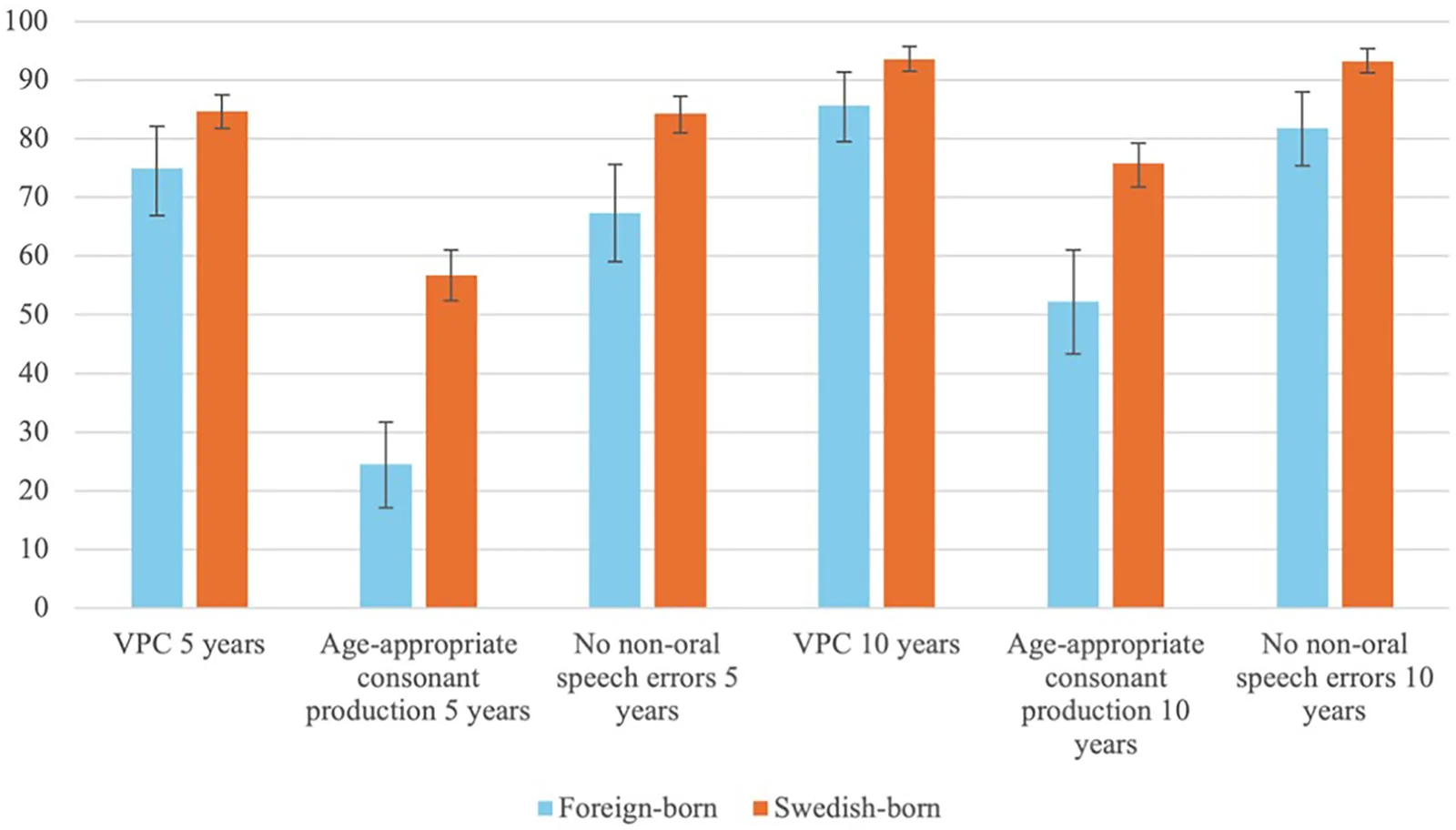
Proportions, with 95% confidence intervals, of positive speech outcomes among foreign-born and Swedish-born children at 5 and 10 years of age. VPC, velopharyngeal competence.

Despite these overall improvements, longitudinal changes in speech outcomes between 5 and 10 years differed significantly between foreign-born and Swedish-born children for all three speech variables (Table 5). Foreign-born children more frequently demonstrated stable poor VPC compared with Swedish-born peers (10% vs 3%), while fewer foreign-born children (71% vs 81%) maintained stable good VPC. Similarly, a higher proportion of foreign-born children had stable poor consonant production (43% vs 19%), and fewer maintained stable good consonant production (20% vs 52%). For non-oral speech errors, a higher proportion of foreign-born children had stable poor outcomes (13% vs 5%) and a lower proportion had stable good outcomes (62% vs 83%) compared with Swedish-born peers (Table 5).

**Table 5.** Changes in Speech Outcomes from Age 5 to 10 in Foreign-Born and Swedish-Born Children.

	n	Stable poor n (%)	Deterioration n (%)	Improvement n (%)	Stable good n (%)	*p*-Value	Effect size
VPC change	714					.002	0.146
Foreign-born	140	14 (10)	6 (4.3)	21 (15)	99 (70.7)		
Swedish-born	574	17 (3)	20 (3.5)	71 (12.4)	466 (81.2)		
Consonant production change	687					<.001	0.273
Foreign-born	130	56 (43.1)	6 (4.6)	42 (32.3)	26 (20)		
Swedish-born	557	107 (19.2)	28 (5)	134 (24.1)	288 (51.7)		
Non-oral speech errors change	692					<.001	0.207
Foreign-born	132	17 (12.9)	7 (5.3)	26 (19.7)	82 (62.1)		
Swedish-born	560	30 (5.4)	8 (1.4)	58 (10.4)	464 (82.9)		

Comparisons between groups were performed using Pearson χ^2^-test with reported frequency, percentage, and associated *p*-value and effect size (Cramér's V). VPC, velopharyngeal competence.

## Discussion

Foreign-born children had poorer speech outcomes at both 5 and 10 years compared with their Swedish-born peers. These findings align with previous studies of IA children with CP ± L, which have reported poorer speech outcomes than in non-adopted peers at 5,^9,10,25,26^ 7–8^9,27^ and 10 years of age.^26^

At both ages, differences remained significant in binary logistic regression models after adjustment for cleft type, additional diagnosed conditions, and sex (Model B). At 5 years, after further adjustment for age at completed primary palatal surgery, the differences persisted for articulation outcomes, whereas the difference observed in VPC was no longer significant (Model C). This suggests that the observed difference in VPC between foreign-born and Swedish-born children at age 5 years can be explained by age at completed primary palatal surgery, consistent with previous findings.^8^ By 10 years, differences in all speech outcomes diminished after adjustment for age at completed primary palatal surgery, suggesting that older age at completed primary palatal closure—rather than being foreign-born—accounted for the observed differences (Model C).

### Speech Development from 5 to 10 Years

The following section describes the longitudinal development of speech outcomes from 5 to 10 years based on chi-squared-test, without adjustment for other variables. Significant differences in longitudinal speech development between foreign-born and Swedish-born children were observed. Both groups showed improvement in all speech outcomes over time, yet the gap between them persisted. Stable poor VPC was 3 times as common among foreign-born children, while stable poor outcomes for consonant production and non-oral speech errors were about twice as common. In addition, a smaller proportion of foreign-born children achieved stable good outcomes compared to their Swedish-born peers. Although a higher proportion of foreign-born than Swedish-born children showed improvement in consonant production and non-oral speech errors, they remained at a disadvantage by 10 years of age, with nearly half still lacking age-appropriate consonant production. This pattern is comparable to findings from a recent longitudinal study of Caucasian children with non-syndromic UCLP from the Scandcleft project.^18^ In that study, speech proficiency was assessed using the composite score for VPC (VPC-Sum) and PCC. At 10 years of age, 44% of children performed below peer level in speech proficiency, most often due to low PCC scores alone or in combination with a VPC-Sum below peer level. Speech proficiency improved between 5 and 10 years, with 23% of children at peer level at 5 years compared to 56% at 10 years. Poor speech proficiency at 5 years predicted speech below peer level at 10 years. Among those below peer level at 5 years, 58% improved to peer level in PCC and 69% in VPC-Sum by the age of 10 years.^18^ These results are not directly comparable to ours because we included all children rather than only those with poor outcomes at 5 years, and different measures of VPC were used. In our study, some children showed deterioration in speech outcomes between 5 and 10 years. Similarly, Persson et al^18^ reported that 12% of children declined in PCC and 14% in VPC-Sum from peer level at 5 years to below peer level at 10 years. Some of the longitudinal differences observed in our study may be influenced by the cut-off used for age-appropriate consonant production, defined as a PCC score within 2 SDs of normative data, corresponding to 86% at 5 years and 92.3% at 10 years. Thus, a child improving from 87% to 91% PCC would nevertheless be classified as having a deteriorated outcome. Furthermore, given the 5-year interval between speech registrations, some changes in speech development may not have been captured. For instance, velopharyngeal dysfunction can emerge after the age of 5 years^18^ as children grow but may be remedied with speech-improving surgery, resulting in VPC at 10 years.^24^ Finally, because the outcome measures were binary, some children with results near the threshold could have been classified differently at the two ages due to variations in assessment across speech-language pathologists. These factors may partly explain some of the longitudinal changes observed across all speech outcomes.

### Age at Completed Primary Surgery

There was a significant difference between foreign-born and Swedish-born children in age at completed primary palatal surgery, with a mean age of 2.4 years for foreign-born children and 1.5 years for Swedish-born children. Foreign-born children most frequently underwent closure between 25 and 36 months (41%), whereas 80% of Swedish-born children had surgery before 25 months. Palatal closure after 25 months was approximately 3 times more common among foreign-born children, while surgery before 18 months was more than 4 times as frequent among Swedish-born children. These findings are consistent with previous studies reporting later primary palatal surgery in IA children than in their non-adopted peers.^9,10,41^ The delayed timing likely reflects age at arrival in Sweden, as some children may have arrived with no or partial palatal surgery, requiring additional procedures after arrival. Unless foreign-born children arrive early, they are unable to follow the Swedish CLP care program from the outset. Access to information on age at arrival and prior surgery would have enabled more detailed comparisons within this group.

### Secondary Intervention

Secondary palatal surgery was approximately twice as common among foreign-born children across all age intervals. This finding is consistent with previous studies reporting higher rates of secondary surgery in IA children than in their non-adopted peers.^10,41^ By contrast, Okhiria et al^26^ found no difference between IA and non-adopted children with UCLP and BCLP in receiving speech-improving surgery. However, that study included only 23 children per group from a single center, which may have influenced the results.^26^

Similarly, speech-language therapy was more common among foreign-born than Swedish-born children across all age intervals. Unfortunately, data on the number of speech-language pathologist visits, including those reported from local speech-language pathologists, were often incomplete and therefore could not be reliably analyzed. One previous study comparing IA and non-adopted children with UCLP and BCLP before 5 years of age and between 5 and 10 years reported no difference in the mean number of speech-language pathologist visits,^26^ although the small, single-center sample may have affected the findings.

Despite higher rates of secondary palatal surgery and speech-language therapy before 5 years and between 5 and 10 years, a greater proportion of foreign-born children had speech difficulties at both 5 and 10 years. This likely reflects greater severity of speech difficulties rather than a lack of treatment effect. Children with more severe speech difficulties are more likely to receive speech-language therapy and/or secondary palatal surgery and still experience residual difficulties at 10 years of age. Similar trends have been noted previously, where 5-year-old children with a higher number of speech-language pathologist visits often had poorer velopharyngeal function^15^ and PCC scores.^3^ Although they may achieve higher PCC scores by 10 years, they remain less likely to reach age-appropriate peer levels.^18^ It is therefore important to consider both age-appropriate levels and clinically relevant improvements, as these are relevant in everyday communication but is not always captured by statistical significance.^50^ The review and meta-analysis by Sand et al^50^ showed that many children who had received speech-language therapy, particularly those under 6 years of age, achieved clinically relevant improvements, and some even reached age-appropriate levels. From this perspective, some children with persistent difficulties at 10 years may nonetheless have made meaningful progress in their speech, though such improvements could not be detected within the constraints of this registry-based study.

### Additional Diagnosed Conditions

Children with additional diagnosed conditions had lower odds of achieving good outcomes across several speech measures. For VPC, children with additional diagnosed conditions had significantly lower odds at 5 years but not at 10 years (Model C), contrasting with Schaar Johansson et al,^24^ who found lower odds of VPC at 10 years among Swedish-born children with CP ± L and additional conditions. Other studies have reported mixed results at age 5 for velopharyngeal function—some found no differences between children with and without Robin sequence,^32,33^ while others reported poorer outcomes among children with Robin sequence^31^ and among those with additional conditions.^28^

Children with additional diagnosed conditions were less likely to achieve age-appropriate consonant production at both ages (Model C), consistent with previous research involving 5-year-olds with additional conditions^8,28,34^ and with Robin sequence.^31,32,34^ Similarly, in a study by Butterworth et al,^51^ 5-year-old children with CP ± L who had two or more additional congenital differences but no diagnosed syndrome had lower odds of achieving good articulation and of having no structurally related speech difficulties. However, Butterworth et al^52^ also found that 5-year-old children with CP and Robin sequence were less likely to have good articulation and no structurally related speech difficulties than those without Robin sequence, though this difference was no longer significant after adjusting for sex and cleft extent. Regarding no non-oral speech errors, children with additional diagnosed conditions had lower odds at 5 years, but not at 10 years (Model C).

This inconsistencies between studies may partly reflect differences in how “additional conditions” are defined and grouped. In the present study, the definition included syndromes, Robin sequence, additional malformations, and/or intellectual disability, whereas other studies excluded intellectual disability.^24,28^ By contrast, Butterworth et al^51^ included children with additional congenital differences but excluded those with diagnosed syndromes. Furthermore, some studies focused solely on children with Robin sequence, all of whom had CP.^31–33^^,52^ These definitional and methodological variations make direct comparison across studies difficult. Additionally, the use of overlapping cohorts in several registry-based studies^8,24,34,41^ may contribute to similarities in reported results.

### Cleft Type and Sex

At 5 years, children with BCLP had 33% lower odds of VPC than did children with UCLP, though this difference was not statistically significant (Model C). By 10 years, children with BCLP had 61% lower odds than to those with UCLP, and the difference was statistically significant (Model C). These results suggest that the impact of BCLP on VPC becomes more pronounced over time, although the relatively small number of children with BCLP (n = 113 in Model C) should be considered when interpreting these findings. This contrasts with Schaar Johansson et al,^24^ who found no association between cleft type and VPC among Swedish-born 10-year-olds, but aligns with results observed at 5 years.^13,32,53^

Children with BCLP were significantly less likely to achieve age-appropriate consonant production at both ages, whereas children with CP showed higher odds than those with UCLP (Model C). These findings align with previous research demonstrating that more extensive clefts, particularly BCLP, are associated with poorer articulation outcomes.^13,32,52–55^ In our sample, both BCLP and UCLP were nearly twice as common among foreign-born children than among Swedish-born children, while CP was almost 5 times more prevalent among Swedish-born children. This difference in cleft type distribution should be considered when interpreting group differences in the results. Similarly, children with BCLP were less likely to have no non-oral speech errors (Model C), consistent with previous findings.^13,53^

Moreover, in agreement with earlier large-sample studies,^8,13^ sex did not significantly affect VPC (Model C). However, boys were significantly less likely to achieve age-appropriate consonant production at both ages then were girls (Model C). This finding is consistent with previous studies involving large cohorts of around 400 or more children, including 5-year-olds with CP ± L^13,52^ and UCLP,^3^ as well as 10-year-olds with UCLP.^16^ Furthermore, boys had lower odds of having no non-oral speech errors at 5 years (Model C), which contrasts with Klintö et al,^13^ who reported no sex difference. This discrepancy may reflect the larger sample size in the present study, which included nearly 300 more children than that of Klintö et al.^13^

### Timing of Primary Palatal Surgery

In this study, higher age at completed primary palatal closure was consistently associated with poorer speech outcomes (Model C). Closure after 25 months was linked to lower odds of VPC at both 5 and 10 years and to lower odds of age-appropriate consonant production at 10 years. For consonant production at 5 years, closure at 10–13 months and after 18 months was associated with poorer outcomes compared with surgery before 10 months. Surgery after 13 months was linked to lower odds of having no non-oral speech errors at 5 years, and surgery after 36 months was associated with lower odds at 10 years. These findings align with previous results of studies showing that delayed palatal closure negatively affects speech outcomes, although the exact age thresholds vary—for example, after 25 months^13^ and after 36 months.^3^ Importantly, when age at completed primary palatal surgery was included in the fully adjusted regression model, the observed differences in all three speech outcomes between foreign-born and Swedish-born children at 10 years were no longer significant (Model C). This suggests that the poorer speech outcomes observed in foreign-born children at 10 years of age were explained by later timing of completed palatal surgery, rather than being foreign-born per se.

### Strengths and Limitations

A key strength of this study was the inclusion of nearly a complete national cohort of children with CP ± L, which allowed investigation of subgroups often underrepresented or excluded in previous research, such as foreign-born children, and those with additional diagnosed conditions and/or BCLP. The longitudinal design is another strength because it enabled the examination of changes in speech over time and identification of factors influencing long-term outcomes.

However, several limitations should be acknowledged. As a registry-based study, the number of available variables was limited. Several potentially important variables that may influence speech development and surgical management were either inaccessible or missing. These include detailed information on age at arrival, surgeries performed abroad, medical history from other countries,^56^ cleft width,^57^ type of speech-language therapy,^50^ number of speech-language pathologist visits, hearing status,^58^ language background, co-occurring speech or language disorder,^59^ and surgeon experience.^60^ Access to such data would have strengthened the adjusted models and provided greater insight into factors affecting speech outcomes.

Assessments were made by single cleft team speech-language pathologists, rather than by several blinded raters with reported intra- and interrater reliability. Although previous research indicates good reliability for binary speech quality indicators,^37–39^ this remains a methodological limitation. Furthermore, the binary nature of the variables yields relatively coarse data, limiting the ability to detect more subtle or potentially clinically meaningful improvements.^50,61,62^

Among the excluded children, a higher proportion were foreign-born, which may partly reflect later arrival to Sweden, in some cases even after 5 years of age. The proportion with additional diagnosed conditions was also higher, as expected, because some of these children likely had no or very limited speech. This may also partly explain the higher prevalence of CP^28–30^ among the excluded participants.

Finally, although this study included a large overall sample, some subgroups were small, particularly among foreign-born children. Therefore, cleft of the soft and hard palate and cleft of the soft palate were merged into CP for statistical analyses. Clinically, differentiating between cleft of the soft and hard palate and cleft of the soft palate can be challenging, and classification has been shown to vary across CLP centers.^13^ While earlier studies suggest that children with cleft of the soft palate may have better speech outcomes than those with cleft of the soft and hard palate,^52,53^ the small number of foreign-born children in each subgroup made separate analyses impractical. Similarly, IA and non-adopted foreign-born children share key characteristics—such as being born outside Sweden and potentially having undergone surgery abroad—but may differ in other aspects, including pre-arrival environment, family setting, and language exposure. IA children experience a new family and often a change in language, whereas non-adopted foreign-born children remain with their biological families and retain their native language. Combining these groups enabled more robust statistical analyses but limited the ability to explore potential differences within this heterogeneous foreign-born population.

### Future Research

Future research should prioritize detailed longitudinal documentation of speech-language therapy, including type and intensity, as the current registry data include limited information on speech-language therapy. More detailed data would enable analyses of which speech-language therapy strategies are associated with clinically meaningful improvement in speech.^50^ The poorer outcomes observed among foreign-born children, despite higher rates of secondary palatal surgery and being registered as having received speech-language therapy at a binary level (yes/no) compared with their Swedish-born peers,^8^ further highlight the need for improved data capture to inform effective intervention strategies. Including foreign-born children in future research is important because they remain underrepresented despite frequently experiencing speech difficulties. Longitudinal study designs are valuable for capturing both age-appropriate outcomes and clinically meaningful progress over time.

### Clinical Implications

The findings emphasize the importance of prioritizing primary palatal surgery soon after arrival for foreign-born children with unoperated cleft, as surgery performed at an older age is associated with lower odds of good speech outcomes. This raises an important clinical and ethical dilemma regarding surgery in IA children with CP ± L. At some CLP centers, primary palatal surgery may sometimes be postponed for psychological reasons—such as allowing children more time to form secure attachments with their caregivers before undergoing surgery. While this may support attachment, our results, consistent with previous findings,^8,49^ suggest that postponing surgery can come at the cost of speech development. Clinicians must therefore carefully balance the potential benefits of attachment development against the risk of long-term speech difficulties.

Although improvements occur over time, a substantial proportion of children still present with speech difficulties at 10 years of age, highlighting the need for continued follow-up and intervention into adolescence to avoid unnecessary delays in secondary palatal surgery or speech-language therapy. Children at higher risk of persistent velopharyngeal dysfunction and speech difficulties—such as those with higher age at primary palatal surgery,^3,13^ BCLP,^13,54^ and/or additional diagnosed conditions^28,31,34^—should be closely monitored to ensure timely and appropriate secondary interventions, including speech-language therapy. For children with velopharyngeal dysfunction, secondary palatal surgery should be considered promptly when indicated to avoid further delays. Given that immigration and international adoption occur globally, these findings are likely generalizable to similar settings where children arrive from abroad without completed palatal surgery.

## Conclusions

Foreign-born children with CP ± L demonstrated poorer speech outcomes at both 5 and 10 years compared with their Swedish-born peers. At 10 years, these differences were associated with higher age at completed primary palatal surgery within the foreign-born group. Despite overall improvement with age, a higher proportion of foreign-born children continued to exhibit poor speech outcomes at 10 years, and nearly half still lacked age-appropriate consonant production. In addition, a greater proportion of foreign-born children underwent secondary palatal surgery and received speech-language therapy. These findings underscore the importance of early primary palatal surgery and ongoing follow-up and intervention into adolescence to optimize long-term speech outcomes.

## Data Availability

The data used in this study are not publicly available due to ethical restrictions but can be obtained from the corresponding author upon reasonable request.
